# Impact of hydrotalcite phases on the texture characteristic of OPC and its correlation with compressive strength and gamma Attenuation

**DOI:** 10.1038/s41598-025-33998-1

**Published:** 2026-02-03

**Authors:** M. Ramadan, M. S. Amin, Alaa Mohsen

**Affiliations:** 1 Chemistry Department, Faculty of Science, Ain Shams University, Cairo, Egypt; 2 Chemistry Department, College of Science, Taibah University, Al-Madinah Al-Munawwarah, P.O. Box 344, Taibah, Saudi Arabia; 3 Faculty of Engineering, Ain Shams University, Cairo, Egypt

**Keywords:** Hydrotalcite, Fresh properties, Compressive strength, Texture parameters, Radiation shielding, Engineering, Materials science

## Abstract

Hydrotalcites (HTs), recognized for their eco-friendly synthesis, layered structure, and exceptional ion-exchange capacity, offer significant potential as functional additives in cementitious systems. Most previous studies used different types of HT based on varying the kinds of di- or tri-valent cations during the preparation process, but they did not change the ratio between them. Accordingly, this study tailored three Mg-Al-CO₃-based HTs with varying Mg: Al ratios (1:1, 2:1, and 3:1, designated HT1, HT2, and HT3) and incorporated them at 1 wt% into ordinary Portland cement (OPC) pastes. The effects of HTs on setting time, workability, and compressive strength were evaluated. Phase composition, textural properties, and microstructure of reference and HT-modified pastes were characterized using XRD, BET/BJH analyses, and SEM/EDX. Additionally, gamma-ray shielding performance against Cs-137 (661.64 keV) was assessed by determining the linear attenuation coefficient (µ) and half-value layer (HVL). Results reveal that HTs accelerate setting, slightly reduce workability, enhance compressive strength, and significantly improve radiation shielding. Among the tailored HTs, HT1 exhibited superior performance, achieving the highest compressive strength (88.8 MPa at 28 days) and greatest shielding efficiency, with µ increased by 112.5% and HVL reduced by 52.9% compared to OPC. These improvements are attributed to HT1’s high surface area, amorphous/mesoporous nature, and its role as a nucleation site and filler, leading to a dense microstructure. Furthermore, incorporating HTs provides an environmentally sustainable approach for producing high-performance cementitious materials with enhanced mechanical and radiological properties, supporting industrial applications in construction and nuclear safety.

## Introduction

Recently, radiation technology has become one of the fundamental indicators by which the progress of countries is measured due to its uses in many fields^1^. Nuclear plants are considered green electricity generators with limited CO_2_ emission^2^. In medical applications, nuclear technology is used in the diagnosis and therapy of diseases and the production of radiopharmaceuticals^3^. In industry, it is used in the radiography process that allows non-destructive testing in the construction, automotive and aerospace sectors^4^. Furthermore, nuclear technology is employed in food production, desalination of water, controlling pollution, manufacturing processes, archeology and military^5^. The increasing utilization of nuclear technology in various sectors causes a great need for protection against hazards of radiation to protect humans and the environment from their detrimental impact^6^.

The efficacy of radiation shielding mainly depends on using the proper materials^7^. Generally, lead, nano-based composites, metallic alloys, glass doped with metal oxide and concrete are employed as radiation shields^8^. Among these materials, concrete is considered the popular choice for shielding against hazardous radiation due to its low production and maintenance costs, versatility and durability^9^. The density of concrete is the radiation shielding controller. Increasing density refers to superior radiation shielding, as the high-density material prevents passing radiation^10^. Accordingly, heavy-weight aggregates such as magnetite, limonite, barite, and goethite are used in the production of concrete^11^. Using such aggregates increases the cost of production^12^, as well as negatively affects the mechanical-performance of concrete^11^.

To overcome the limitations of using heavy-weight aggregates, a new generation of shielding concrete is produced by embedding nano and mesoporous particles in the cement matrix. The high surface area of these particles improves mechanical-performance via acting as filler for the microporous in the cement matrix and nucleation seeds for the formation of hydration products^13–15^. In addition, enhancing the mechanical-properties by densifying the microstructure filling the porous increases the probability of interaction with radiation, improving the attenuation coefficient^6^. Several previous studies prove that the incorporation of nano-Fe_2_O_3_^16^, nano-ZrO_2_^17^, nano-TiO_2_^18^, nano-ZnO^19^, nano ZnFe_2_O_4_^19^, nano-Cr_2_O_3_^20^, nano-PbO^6^, nano-PbTiO_3_^6^, nano-Bi_2_O_3_^21^, nano-NiO^22^, mesoporous-WO_3_^23^, mesoporous-MgO^24^, microporous-Al_2_O_3_^25^ results in improving the radiation shielding while maintaining or improving the mechanical-properties.

Lately, layered-double hydroxides (LDHs) have been given great attention in many fields. HT is one of the prominent representative families of LDHs with a general formula [M^2 +^_1-x_M^3+^_x_(OH)_2_](A^n-^)_x/n_.zH_2_O, where M^2+^ and M^3+^ are di- and tri-valent metal cations (such as Mg^2+^ and Al^3+^), A is an n-valent anion (such as CO_3_^2-^)^26^. The easy, fast, cheap and green methods followed in the preparation of HTs^27^, as well as their unique fine-tunable structure and their high ability to exchange anions^28^, make this family a promising material for several applications. HTs are used in the chemical industry, polymer production, CO_2_ capture, pharmaceutical industries and in construction materials^27^.

Several studies have tried to use HTs’ high surface area, easy handling structure (tailor-made), and anion-exchanging capacity to improve the properties of concrete and add new features to it. Yang et al. modified the chemical composition of commercial Mg_2_Al-CO_3_-based HT (Mg: Al = 2:1) by using sodium nitrite and sodium p-aminobenzoate to produce Mg_2_Al-NO_2_ and Mg_2_Al-pAB, respectively. They studied the impact of replacement cement with two doses (5 and 10 wt%) from these compounds on the strength and chloride ingress in cement-mortar. It was detected that 5 wt% Mg_2_Al-NO_2_ or Mg_2_Al-pAB cause remarkable enhancement in chloride-diffusion resistance with a slight negative impact on the mortar’s strength. Also, the results showed that Mg_2_Al-pAB is more preferred than Mg_2_Al-NO_2_^29^. Yang et al. evaluated the durability of cement mortar modified with Mg_2_Al-NO_2_ and Mg_2_Al-pAB by following the corrosion of reinforced steel. The data revealed that incorporating 5 wt% Mg2Al-pAB doubled the time-to-corrosion initiation compared to the reference specimen (without HT). From this, it can be concluded that Mg-Al-based HT enhances the durability of construction materials^28^, like geopolymer^30,31^. Li et al. studied the effect of different HTs containing various laminate elements on the properties of sulfoaluminate cement; they prepared ZnAl-HT (Zn: Al = 2:1), MgAl-HT (Mg: Al = 2:1) and ZnMgAl-HT (Zn: Mg: Al = 1:1:1). They found that MgAl-HT and ZnMgAl-HT increase the heat of hydration, in contrary with ZnAl-HT. Adding 1 wt% ZnAl-HT reduces the compressive-strength up to 1-day due to releasing of Zn ions that retard the hydration process. At 7 and 28-days, the specimens containing 1 wt% MgAl-HT has compressive-strength values higher than others containing ZnMgAl-HT than ZnAl-HT than without HT (reference). The nucleation effect and synergistic impact of releasing zinc, magnesium and carbonate ions affected the mechanical-performance^32^. Guan et al., prepared Mg_3_Al-CO_3_-based HT (Mg: Al = 3:1) by the coprecipitation method, then used it to prepare Mg_3_Al-TA-based HT (Mg: Al = 3:1) using an ion exchange process between carbonate ion and tartaric acid. They studied the impact of the prepared HTs on the thickening time and compressive-strength of oil-well cement. They detected a reduction in the thickening time from 140 min for reference specimen (without HT) to 118 min after adding 0.7 wt% Mg_3_Al-CO_3_-based HT. In the case of specimens containing 0.7 wt% Mg_3_Al-TA-based HT, the thickening time increased to 466 min. Adding 1.0 wt% Mg_3_Al-TA-based HT enhances the compressive-strength by 99.6% for up to 3-days; after that (at 7 and 14 days), the compressive-strength decreased but was still higher than that of the reference specimen^33^. Lozano-Lunar et al. prepared three Zn-Al-based HT containing different groups between its layer structure carbonate (Co_3_^2-^), ethylene-diamine-tetraacetate (EDTA) and dimercapto-succinate (DMSA). They studied their impact on the Pb-immobilization in the cement mortar containing a high ratio from electric-arc furnace dust. The results show that all prepared HT has a negative effect on compressive-strength. Regardless of the type of HT employed, all specimens show a significant reduction in the concentration of leached Pb, reaching a 50% reduction in the case of Zn-Al-DMSA^34^.

Previous literature primarily concentrated on modifying HTs by altering di- or tri-valent cations and interlayer anions to enhance the fresh and hardened properties of cement-based systems. These efforts mainly aim to improve durability, minimize chloride penetration, prevent steel reinforcement corrosion, and immobilize heavy metals for environmental safety. Nevertheless, significant research gaps remain unaddressed: (i) the impact of changing the ratio between di- and tri-valent cations (such as the Mg^2+^/Al^3+^ ratio) on the structural, textural, and functional properties of HTs and their subsequent influence on cement performance remains insufficiently explored; and (ii) investigations into the role of HTs in gamma-ray attenuation within cementitious composites are limited, despite the increasing demand for radiation-resistant construction materials in nuclear and healthcare sectors. Addressing these issues is crucial for creating multifunctional, sustainable cementitious materials that combine superior mechanical performance with enhanced radiological shielding, thus promoting progress in construction technology and environmental responsibility. Therefore, the main objectives of this study are (i) preparing different Mg-Al-CO_3_-based HT compounds by changing Mg^2+^/Al^3+^ ratio (1:1, 2:1 and 3:1). After that studying the impact of these ratios on the degree of crystallinity, crystallite particle sizes, crystallographic structure, morphology and textural parameters of synthesized HT compounds; (ii) evaluating the impact of incorporating the prepared Mg-Al-CO_3_-based HT compounds on the setting time, workability, compressive strength, gamma-ray radiation shielding of cement pastes; (iii) monitoring the alternation in the phase composition, microstructure and pore structure distribution in the hardened paste after inclusion the prepared HTs in the cement system and (iv) discussing the mechanism about the role of Mg-Al-CO_3_-based HT in radiation shielding.

## Materials and methodology

### Materials

Portland cement CEM I 42.5 N with a Blaine surface area of 380 m^2^/kg, grain size below 75 μm and specific gravity of 3.15 from the National Company for Cement in Beni Seuf, Egypt, was used to prepare cement pastes. Its oxide composition was analyzed by X-ray fluorescence (XRF - model PW-1400 - Xios) as in Table 1. Commercial anhydrous aluminium chloride (AlCl_3_), magnesium chloride hexahydrate (MgCl_2_.6H_2_O), sodium carbonate (Na_2_CO_3_), and deionized water are the main precursors used to prepare the HT family. They were purchased from El-Gomhuriya Company, Egypt. A polycarboxylate-based superplasticizer (PCb-SP - ViscoCrete-3425) from Sika Company, Egypt, was used as a dispersing agent for HT compounds in cement pastes.

**Table 1:** Chemical oxide compositions for OPC (mass, %)

Material	SiO_2_	Al_2_O_3_	Fe_2_O_3_	CaO	MgO	SO_3_	Na_2_O	K_2_O	Cl^-^	LOI
OPC	20.69	4.67	3.51	64.11	1.27	2.92	0.29	0.22	0.02	2.30

### Hydrotalcite family Preparation

**Fig. 1:**
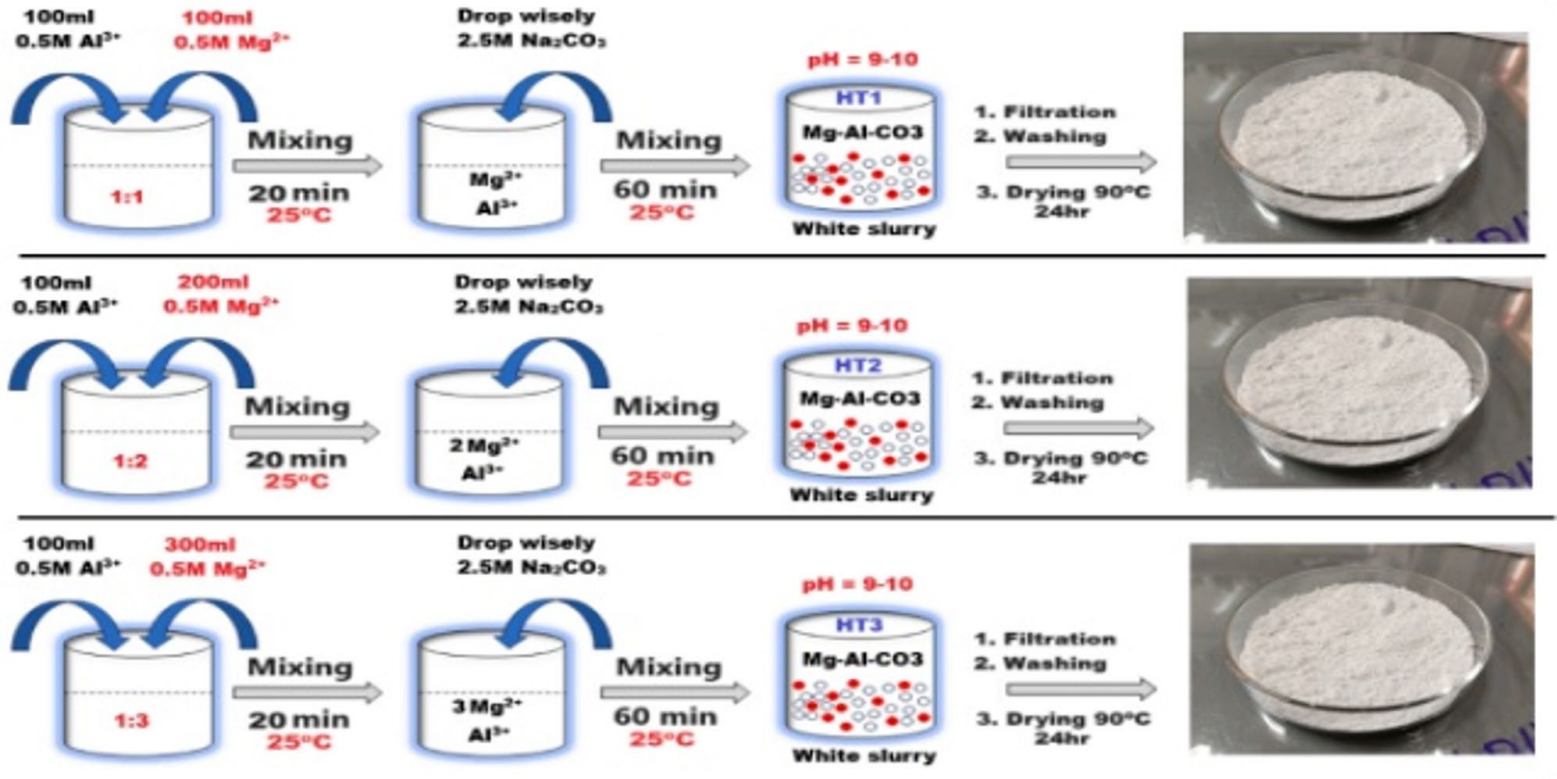
A schematic diagram for the synthesis of hydrotalcites.

Three compounds from the magnesium-aluminium (Mg-Al)-HT family with different Mg: Al ratios (1:1, 2:1, and 3:1, coded HT1, HT2, and HT3, respectively) were prepared via the coprecipitation method^35,36^. Firstly, a 0.5 M solution from AlCl_3_ and MgCl_2_.6H_2_O, as well as a 2.5 M solution from Na_2_CO_3_, were prepared. In HT1, HT2 and HT3, 100 ml 0.5 M AlCl_3_ was mechanically mixed with 100, 200 and 300 ml 0.5 M MgCl_2_.6H_2_O, respectively, for 20 min at 25 °C to obtain a homogenous solution. After that, the 2.5 M Na_2_CO_3_ solution (precipitating agent) was added dropwise to the three prepared mixed chloride solutions until the P^H^ reached 9–10. The mechanical mixing continued for a further 60 min at 25 °C. Later, the former white slurries (HT1, HT2 and HT3) were aged for 24 h, then filtered and washed with deionized water several times to remove Na^+^ and Cl^−^ ions. Finally, the filtered white slurries were dried at 90 °C for 24 h. After that, the dried powders were ground in a Planetary ball mill (LABTRON - LPBM-A10) and then passed through a 200-mesh sieve. A schematic diagram for the preparation procedure is represented in Fig. 1.

The synthesized HTs (HT1, HT2, HT3) were comprehensively characterized using X-ray diffraction (XRD), Brunauer-Emmett-Teller (BET) and Barrett-Joyner-Halenda (BJH) models, and scanning-electron microscope connected with X-ray analyzer (SEM/EDX) techniques to evaluate their phase composition, texture parameters and morphological properties, respectively. XRD analysis was conducted on a PANalytical X’Pert-Pro diffractometer with Cu Kα radiation (λ = 1.5406 Å) at 40 kV and 40 mA, scanning from 5° to 70° (2θ) with a 0.02° step size to identify crystalline phases and estimate crystallite size. Specific surface area and meso-porosity were determined via nitrogen adsorption–desorption isotherms at 77 K using a Belsorp Mini-x analyzer after degassing samples at 120 °C for 6 h. The BET model was applied within a relative pressure range of 0.05–0.30 (P/P₀) to calculate specific surface area, while the BJH model derived pore size distribution and pore volume from the desorption branch, suitable for mesoporous layered structures. Morphological features were examined using SEM (Thermo Scientific Quatro-S) operated at 15 kV, with samples gold-coated to enhance conductivity. Elemental composition was assessed by EDX integrated with SEM, analyzing three representative spots per sample to determine atomic percentages of Mg, Al, O, and C, which were averaged and reported in Table 3. These combined analyses provided insights into the influence of Mg/Al ratio on crystallinity, textural features, and engineering shapes of the hydrotalcites.

### Cement paste Preparation

To study the impact of prepared HT compounds on the fresh and hardened properties, as well as radiation shielding of cement paste, four specimens were prepared by mixing Portland cement with water containing 0.1 wt% PCb-SP and 0 wt% HT (coded OPC specimen, reference) or 1 wt% HT1, HT2 or HT3 (coded OPC-HT1, OPC-HT2 and OPC-HT3 specimens, respectively). All pastes were prepared using a constant water/binder ratio (W/B ratio = 0.25), as shown in Table 2. The mixing process was performed in a Hobart-mixer for 5 min to obtain workable pastes. The fresh pastes were cast in cubic molds (dimension 2.5*2.5*2.5 cm), vibrated to remove air bubbles and then placed in a humidity chamber with a relative humidity of 98 ± 2% at 25 °C for 24 h. After that, the hardened specimens were demolded and cured under tab-water for 1, 3 and 28-days.

**Table 2:** Mix design and notations of Cement composites (mass%).

Composite	OPC %	HT1%	HT2%	HT3%	W/B %	PCb-SP%
OPC	100	-	-	-	0.25	0.1
OPC-HT1	100	1	-	-	0.25	0.1
OPC-HT2	100	-	1	-	0.25	0.1
OPC-HT3	100	-	-	1	0.25	0.1

### Testing methods

The fresh-properties of prepared cement pastes without/with HT were assessed by measuring the setting time by the Vicat apparatus according to ASTM C191-19^37^ and workability by mini-slump test^38–40^. In the workability test, a high W/B ratio, equal to 0.4, was used to detect the difference in the spread area between pastes.

For mechanical-performance, at each curing time (1, 3 and 28-days), the average compressive-strength value for three cubes from each specimen was measured according to ASTM C109M-20b^41^, using a compression machine (Control, maximum-load equal to 60 tons). The hydration products’ composition, as well as texture parameters and microstructure of cement matrices were assessed using XRD, BET/BJH models and SEM/EDX.

To evaluate the impact of HT compounds on the shielding of hazardous gamma-rays, the linear-attenuation coefficient (µ) was calculated using Beer-Lambert law (I = I⁰e^−µx^) against the Cs-137 isotope, a source of gamma-rays with an intensity of 661.64 KeV. Also, the thickness of the half-value layer was calculated (HVL= -ln(0.5)/µ). The hardened cubic specimens were directed to gamma-rays. The NaI (TI) detector was used to measure the gamma-rays’ intensity in the absence (I⁰) and presence (I) of specimens. Different specimens’ thicknesses (x = 2.5, 5, 7.5 and 10 cm) were used to measure different transmitted intensities (I).

## Results and discussion

### Characterization of synthetic hydrotalcites

**Fig. 2:**
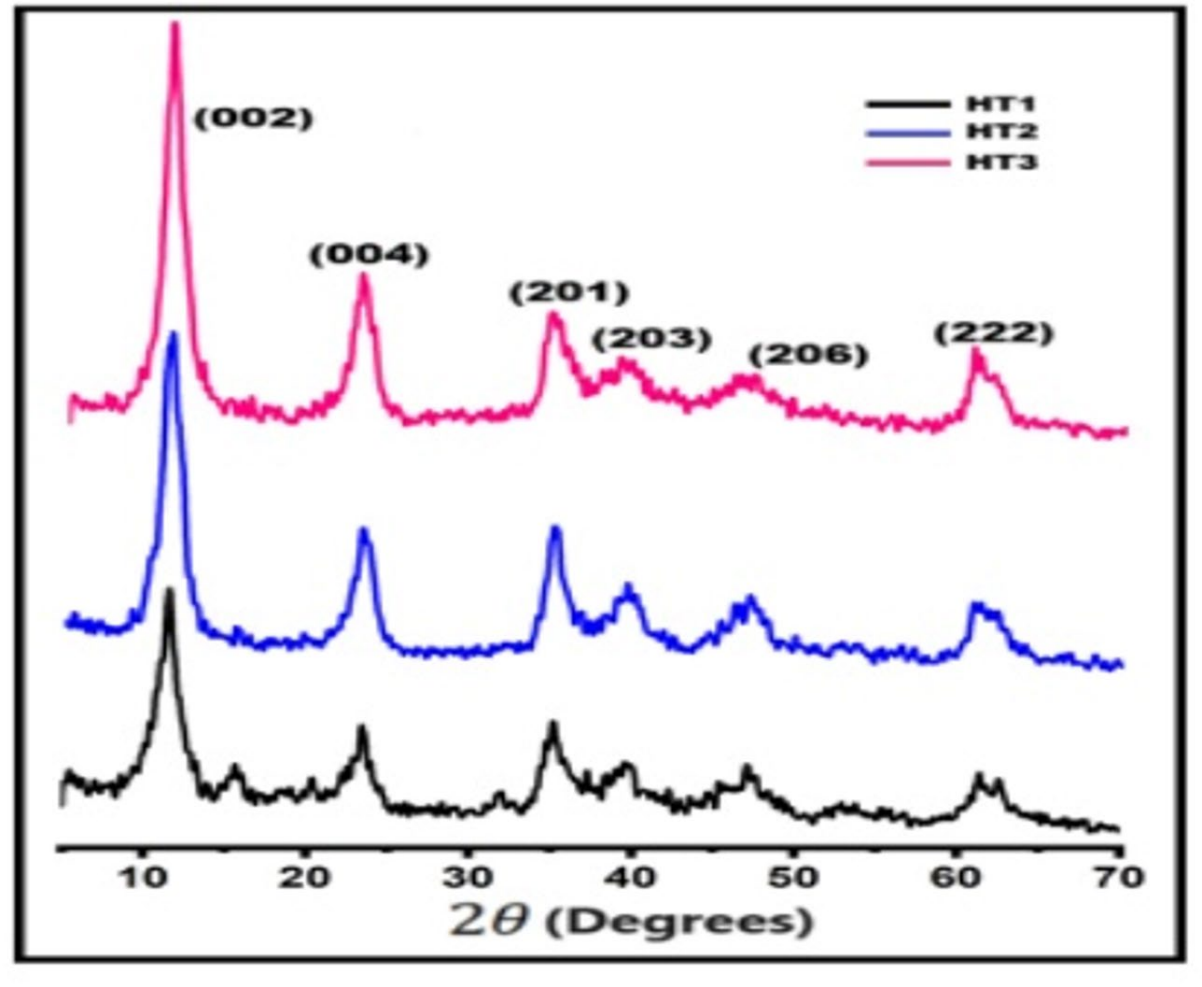
XRD-patterns of synthetic hydrotalcites with different Mg/Al ratios.

The effect of Mg/Al ratio significantly affects the morphological properties, degree of crystallinity and textural parameters of synthesized HT (HT1, HT2, and HT3) as Mg/Al ratios of HT1, HT2, and HT3 are 1, 2, and 3, respectively^35,36^. These changes can be proven through many modern techniques such as XRD, SEM/EDX, and BET/BJH. Figure 2 displays XRD-patterns of different HTs. Despite the use of inexpensive commercial salts (AlCl_3_ and MgCl_2_.6H_2_O), the image confirms that the prepared HTs have a high degree of purity, free of impurities, especially chloride ions. Six main reflections (h, k, l values) are detected at 2θ = 11.47° (002), 23.05° (004), 34.55° (201), 39.05° (203), 46.73° (206) and 60.63° (222). All these distinguished reflections completely match the magnesium aluminum carbonate hydroxy hydrate of three different crystallographic structures. The first one is Mg_6_Al_2_(CO_3_) (OH)_16_·4H_2_O as the crystal system is Rhombohedral (PDF#00–054-1030). The second phase is (Mg_6_Al_2_ (CO_3_) (OH)_16_ 4.5(H_2_O))_0.25_ with hexagonal crystal system (PDF#01–082-8041) while the third phase is Mg_2_Al(CO_3_)(OH)_6_. 0.5·H_2_O (PDF# 00–056-0956, unknown crystal system). As indicated in Fig. 2, increasing the Mg/Al ratio from 1 to 3 caused an increment in the degree of crystallization of HTs. The high dose of magnesium helped form highly crystalline phases, mainly hexagonal and rhombohedral systems^42–44^. According to Match. Program version 3.15, the degrees of crystallization of HT1, HT2 and HT3 are 39.61, 44.56, and 51.03%, while the average crystallite particle sizes are 5.1, 6.9 and 19.1 nm, respectively.

**Fig. 3:**
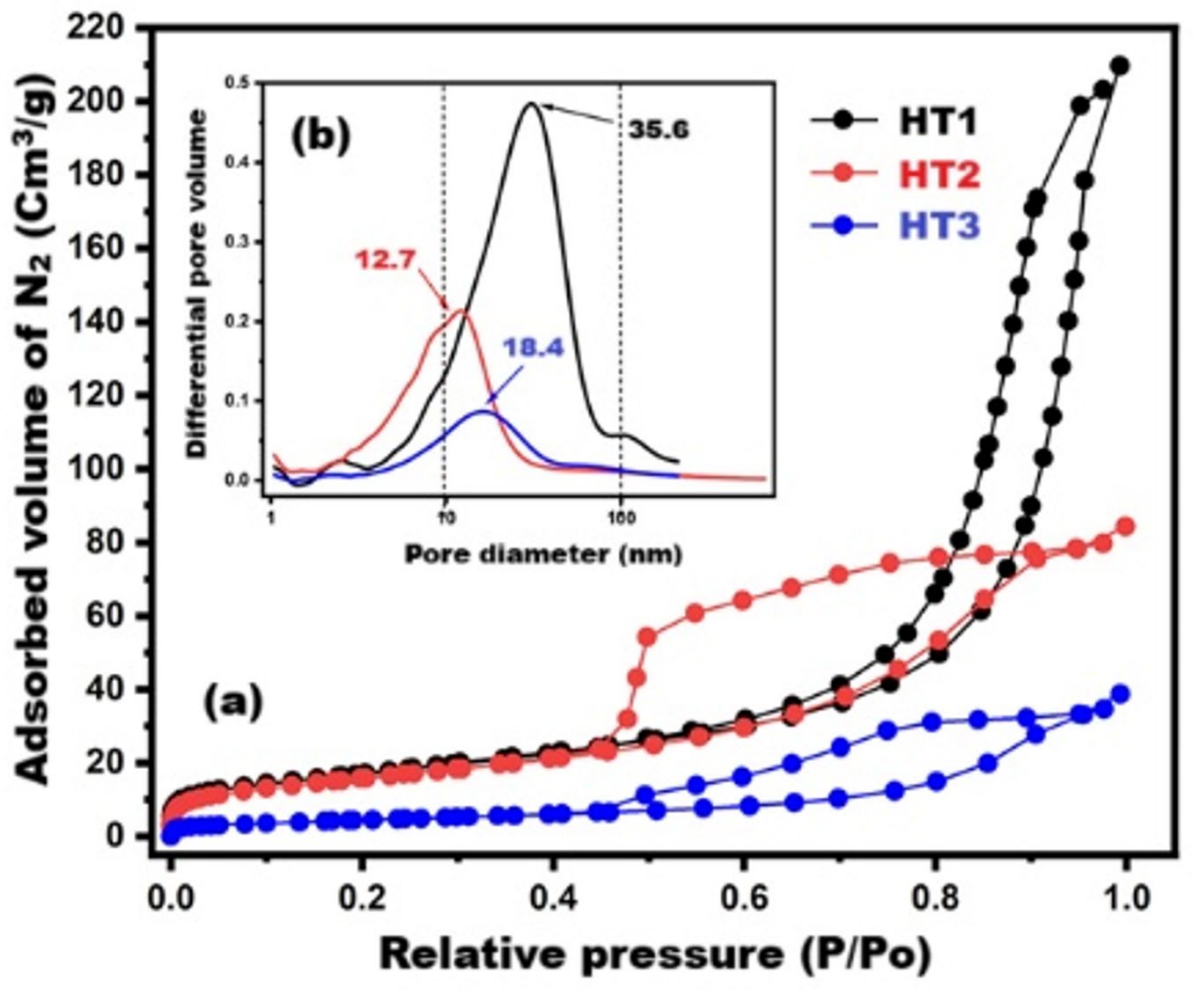
(**a**) adsorption/desorption isotherms and (**b**) BJH -pore size distributions.

Figure 3 illustrates N_2_-adsorption/desorption isotherms and BJH-pore size distributions for the synthetic HTs. Figure 3-a confirms that HT1 (Mg/Al ratio = 1), HT2 (Mg/Al ratio = 2), and HT3 (Mg/Al ratio = 3) match with Type IV isotherm with different hysteresis loops according to IUPAC classification. HT1 adsorbent obeys Type IV with an H3-hysteresis loop, confirming a meso-porous cylindrical system^16^. On the other hand, the other HTs (HT2, HT3) obey Type IV with an H1-hysteresis loop, indicating a meso-porous ink-bottle structure^45^. As clarified in Fig. 3-a, HT1 particles possess the highest adsorbed volume of N_2_ gas, highest adsorption capacity, highest surface area and highest total pore volume. It was found that the specific surface areas (SSA) for HT1, HT2, and HT3 are 63.13, 58.03, and 16.89 m^2^/g, while total pore volumes (TPV) are 0.3209, 0.1288, 0.059 cm^3^/g and monolayer capacities (Vm) are 14.50, 13.33, and 3.88 cm^3^/g, respectively. Therefore, these outputs represent preliminary evidence of the expected good catalytic performance for HT1 and HT2 inside the cementitious reactions^20^. Moreover, BJH-pore size distributions (Fig. 3-b) confirm that the maximum pore diameters for HT1, HT2, and HT3 are 35.6, 12.7, and 18.4 nm, respectively. This data affirms the meso-porosity nature (dpmax = 2–50 nm) for all synthetic HTs.

**Fig. 4:**
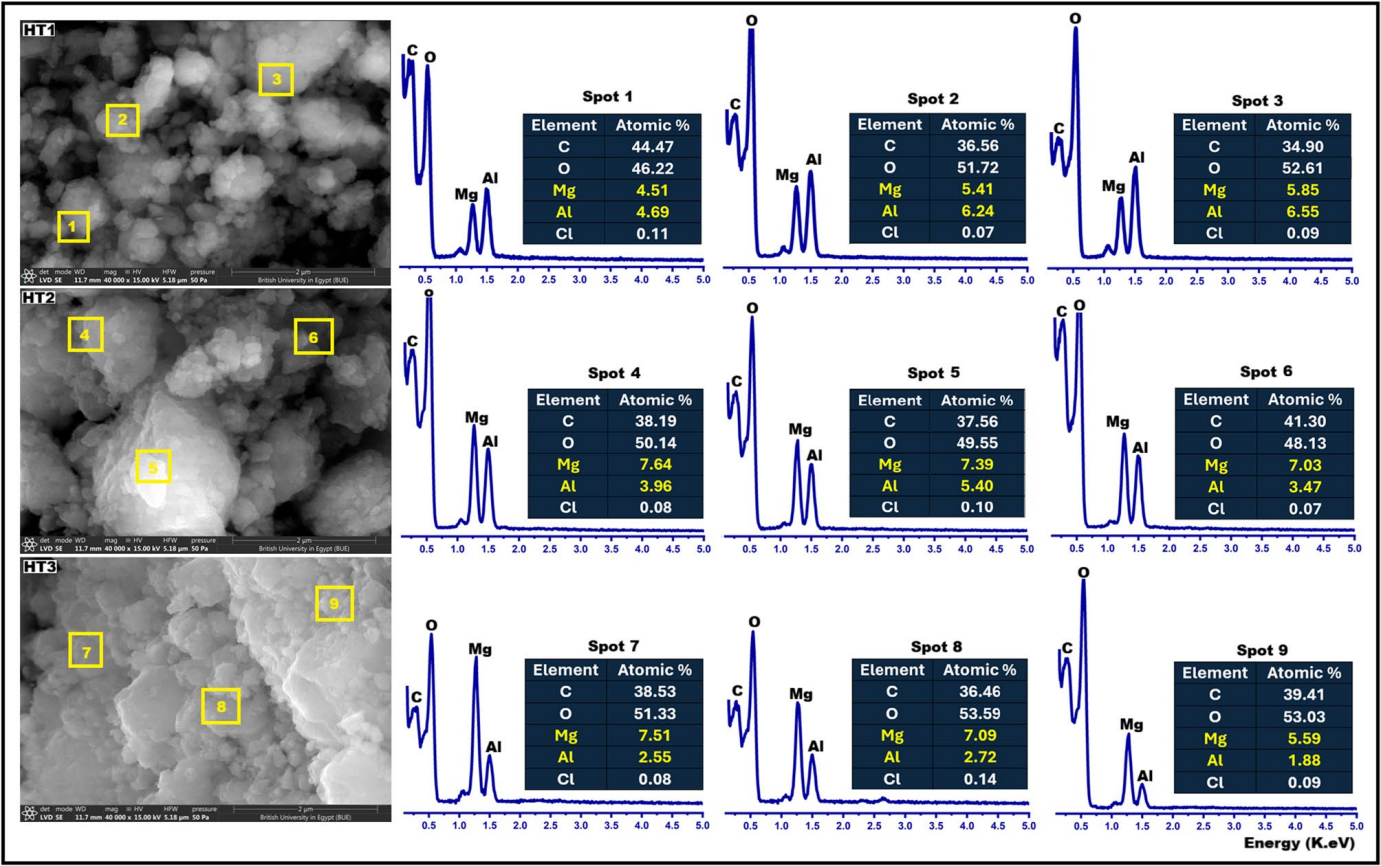
SEM/EDX of synthetic hydrotalcites with different Mg/Al ratios.

Figure 4 presents SEM/EDX micrographs of the synthesized HTs. For each specimen, three representative spots of EDX analysis were taken on the same SEM-image. The microstructural characteristics, morphological features, crystallinity, and average particle size exhibit clear variations among the different compositions. For HT1 (Mg/Al = 1), the morphology is dominated by small, irregularly distributed flakes interspersed with semi-spherical phases, indicative of limited crystal development. Progressive crystal growth is evident as the Mg/Al ratio increases to 2 and 3. In particular, HT3 (Mg/Al = 3) displays well-defined, ultrathin hexagonal plates arranged in stacked layers, characteristic of highly crystalline HT. The EDX spectra confirm the presence of the principal elements (O, C, Mg, Al) in all samples. Despite employing cost-effective commercial precursors (AlCl_3_ and MgCl_2_·6H_2_O), the EDX analysis demonstrates exceptional purity, exceeding 99.84% for all HTs (Mg-Al-CO₃·OH·H₂O). As shown in Table 3, the average atomic percentages of Mg and Al in HT1 are 5.26 and 5.83, respectively, corresponding to an Mg/Al ratio of 0.90. This ratio was insufficient to yield well-ordered HT crystals, resulting instead in a highly amorphous structure. Conversely, HT2 and HT3 exhibit Mg/Al ratios of 1.72 and 2.82, respectively, with the latter confirming the formation of HT in its characteristic crystalline morphology. These SEM/EDX observations are in complete agreement with the XRD findings.

**Table 3:** EDX results for synthetic hydrotalcites with different Mg/Al ratios.

Hydrotalcites	Average atomic %				Average Mg/Al ratio
	C %	O %	Mg %	Al%	
HT1	38.64 ± 5.11	50.18 ± 3.46	5.26 ± 0.68	5.83 ± 0.99	0.90
HT2	39.02 ± 2.00	49.27 ± 1.03	7.35 ± 0.31	4.27 ± 1.00	1.72
HT3	38.13 ± 1.51	52.65 ± 1.17	6.73 ± 1.01	2.38 ± 0.44	2.82

### Fresh properties

**Fig. 5:**
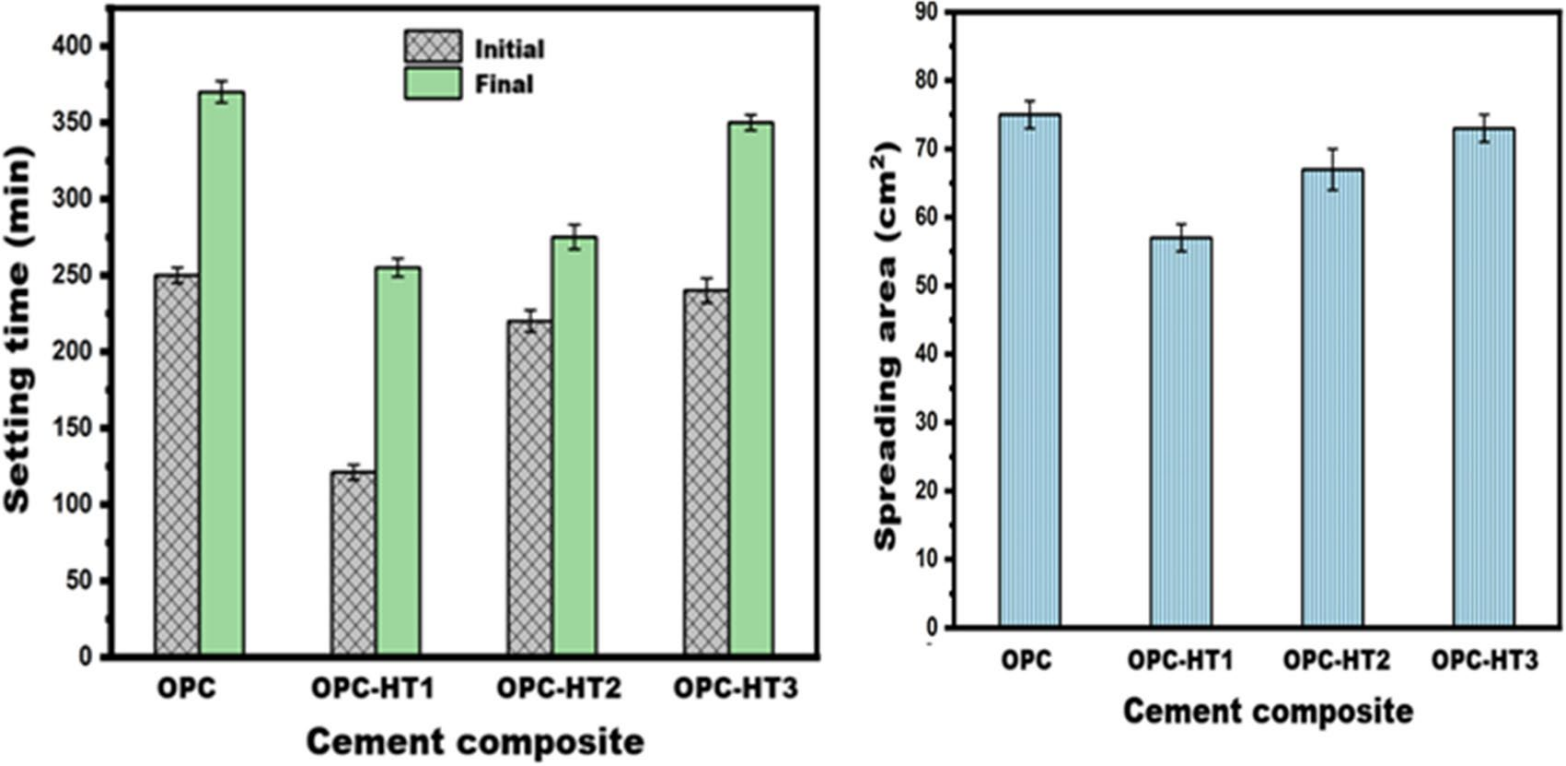
Impact of different types of hydrotalcites on the (**a**) setting time and (**b**) workability of OPC.

Generally, the fresh-properties of cementitious materials affect the ability to handle, compact, and consolidate; therefore, the impact of HTs on setting time and workability was assessed. Figure 5a shows that all specimens without and with HTs have an initial/final-setting time (IST and FST) between 121 and 370 min. This indicates that the I/F-ST is in the acceptable range, according to ASTM C150/C150M-20^46^, confirming their adequate workability during handling and proper curing before removing formwork. It is clarified that HTs cause acceleration in the I/F-ST, which is in line with Wu et al^47^. and Guan et al^33^.. Several reasons may explain this phenomenon. The high surface area of HTs causes more mixing water to be adsorbed, which increases the paste’s stiffness and thus reduces the I/F-ST. The nucleation seeds’ action of HTs accelerates the I/F-ST via lowering the energy barrier required for precipitating hydration products from the pore solution^48^. The filling effect of HTs reduces the pore radii, increasing their compacting rate with hydration products, shortening the I/F-ST^49^. HTs can release Mg^2+^ and Al^3+^ ions into the pore solution, promoting faster precipitation of hydration products such as calcium aluminate hydrate (CAH), which is responsible for the setting of the cement matrix^50^. The immobilization of the SO_4_^2−^ group from the gypsum in the HT structure (LDH) via the ion exchange process may be another reason for the acceleration of the setting. The Mg^2+^ and Al^3+^ ions in the HT structure drive electrostatic attraction for anions. During this process, the original interlayer carbonate (CO_3_^2−^) is replaced by sulfate ions (SO_4_^2−^) originating from gypsum, which become trapped within the HT structure and are thus removed from the pore solution. It is well known that the SO_4_^2−^ anion from the gypsum reacts with C_3_A in the cement matrix to form ettringite, which regulates the setting time. Immobilization of the SO_4_^2−^ group in the HT structure hinders the ettringite’s formation and thus accelerates the setting, as discussed by Wu et al. ^47^. The significant acceleration rate of HT1 with respect to HT2 and HT3 is due to its high surface area.

Regarding workability, Fig. 5b clarifies that OPC-HT1 and OPC-HT2 are less workable than OPC (reference specimen, without HT); their spreading area is 24% and 10.7% lower than OPC, respectively. This matches Long et al^49^.. On the other hand, OPC-HT3 is workable, just like OPC. The amorphous nature and high surface area of HT1 and HT2 concerning HT3 cause adsorbing a portion of mixing water and increase the friction between the matrix’s components, thus hindering the flow of the paste and then decreasing the workability^51–53^.

### Phase compositions of cementitious composites

**Fig. 6:**
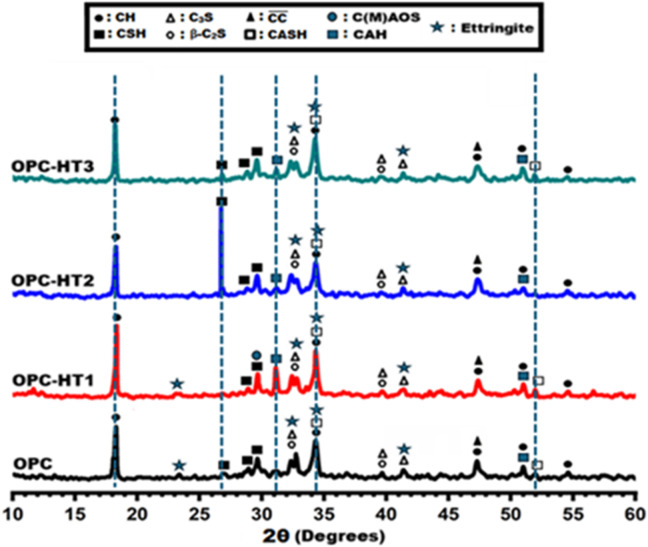
XRD-patterns of OPC pastes modified with different types of hydrotalcites.

Figure 6 shows XRD-patterns of hardened OPC pastes that were grafted with individually 1 wt% of different types of HTs. All fabricated pastes (OPC, OPC-HT1, OPC-HT2, OPC-HT3) were cured for up to 28-days under a normal treatment regime (curing under tap water at 25 °C). XRD-patterns of reference paste (OPC) indicate the formation of various hydration products (CH, CAH, CASH, CSH, ettringite^54,55^, unreacted calcium silicates (C_3_S and $\:\beta\:$-C_2_S)^56,57^, in addition to the monoclinic calcite phase ($\:\stackrel{-}{CC}$, PDF#00–051-1524)^58,59^. The characteristic peaks of Portlandite (CH, hexagonal calcium hydroxide, Ca(OH)_2_, PDF# 01–089-2779) were detected at 2$\:\theta\:$= 18.29, 34.31, 47.27, 50.99, and 54.59^o^. Highly crystalline peaks that distinguish the ettringite phase (Trigonal Al_2_Ca_6_H_66_O_49.68_S_3_) were observed at 23.37, 32.29, 34.55, and 41.51^o^. Specific peaks were observed for ill-crystalline calcium silicate hydrate/Tobermorite (CSH, Ca_2_SiO_4_.0.5H_2_O, PDF# 00–012-0199) at 2$\:\theta\:$= 28.97 and 29.67^o^ while calcium aluminate silicate hydrates (CASH, PDF#00–013-0567 and PDF#00–039-1373) at 2$\:\theta\:\:$=34.27 and 51.99^o^. The later (CASH) possessed two different crystallographic structures, namely as monoclinic gismondine (CaAl_2_Si_2_O_8_·4H_2_O) and orthorhombic lawsonite (CaAl_2_Si_2_O_7_(OH)_2_·H_2_O). Another phase of hydration yield was observed at 2$\:\theta\:\:$= 51.99^o^; that indicates generating cubic hydrogarnet (CAH, 3CaO.Al_2_O_3_.6H_2_O), PDF#96–900-1036). All previous phases are responsible for the mechanical performance of OPC pastes at 28-days.

The inclusion of 1%HT1 to OPC pastes (OPC-HT1) led to some noticeable changes (5 dashed lines), such as (i) an increase in the peak intensity of free lime (CH) and CASH at 2$\:\theta\:$= 18.29 and 34.3^o^, (ii) the appearance of a new peak at 2$\:\theta\:\:$=31.13^o^ that characteristic for hexagonal/cubic calcium-aluminate-hydrates mainly as hydrogarnet, (iii) the formation of a new phase of monoclinic calcium magnesium alumino oxy silicate (C(M)AOS, Ca_54_MgAl_2_Si_16_O_90_, PDF#00–013-0272). Therefore, the addition of mesoporous HT, of one Mg/Al ratio, has played an effective role in stimulating cement reactions to generate extra hydration products. On the other hand, a high crystalline peak was observed at 2$\:\theta\:\:$=26.73^o^, in the case of OPC-HT2 paste, due to crystals growth of CSH (Clino-Tobermorite), while no phase changes occurred in the presence of HT3. Table 4 clarifies the quantitative phase analysis for all OPC-HT pastes using MATCH PROGRAM 3.15. Quantitative XRD analysis offers valuable insights into the influence of hydrotalcite incorporation on cement hydration. Among the examined systems, OPC-HT1 exhibited the lowest residual clinker phases (Alite: 15.9%, Larnite: 3.6%) compared to OPC (Alite: 21%, Larnite: 3.8%) and OPC-HT2 (Alite: 19.4%, Larnite: 3.7%), indicating a pronounced acceleration of hydration. Furthermore, OPC-HT1 demonstrated the highest formation of strength-contributing hydration products, including CSH (24.1% + 1.6%), CASH (14.3% + 11.6%), and hydrogarnet (5.2%), whereas OPC-HT2 and OPC-HT3 yielded comparatively moderate amounts of CSH (15.1% + 6.1%) and (20.6% + 1.6%), respectively. Notably, OPC-HT1 also exhibited the greatest proportion of free lime (12.7%) after 28 days of curing, reflecting its enhanced reactivity. These findings confirm that HT1 (Mg/Al = 1) effectively promotes the transformation of primary silicate phases into stable hydrates. This superior performance is attributed to HT1’s high surface area (63.13 m²/g), substantial amorphous content (~ 60%), and nanometric particle size (5.1 nm), which collectively facilitate rapid nucleation, precipitation of hydration products, and pore refinement. In contrast, HT2 and HT3, characterized by higher Mg/Al ratios, displayed diminished catalytic efficiency, as evidenced by greater residual clinker content and reduced main hydrates (CSHs and CASH) formation. Overall, HT1 not only accelerates hydration but also modifies the phase assemblage toward products that enhance compressive strength and microstructure, underscoring its advantage over HT2 and HT3.

**Table 4:** Semi-quantitative phase analysis (QPA) for the fabricated OPC-HTs pastes.

Composite Paste	Quantitative phase analysis (QPA%)										
	Tobermorite (CSH) %	Clino-Tobermorite (CSH) %	Gismondite (CASH) %	Lawsonite (CASH) %	Hydrogarnet (CAH) %	Portlandite (CH) %	Ettringite (AFt) %	Calcite ($\:\stackrel{-}{\boldsymbol{C}\boldsymbol{C}}$) %	Alite (C_3_S) %	Larnite ($\:\boldsymbol{\beta\:}$-C_2_S) %	C(M)AOS %
OPC	22.1	2.0	14.1	11.1	2.9	11.0	10.0	2.0	21.0	3.8	-
OPC-HT1	24.1	1.6	14.3	11.6	5.2	12.7	7.1	2.2	15.9	3.6	1.7
OPC-HT2	15.1	6.1	16.1	9.2	3.5	11.8	11.9	3.2	19.4	3.7	-
OPC-HT3	20.6	1.6	15.1	8.9	4.5	11.7	9.9	3.9	20.2	3.6	-

### Meso-porosity of cementitious pastes

**Fig. 7:**
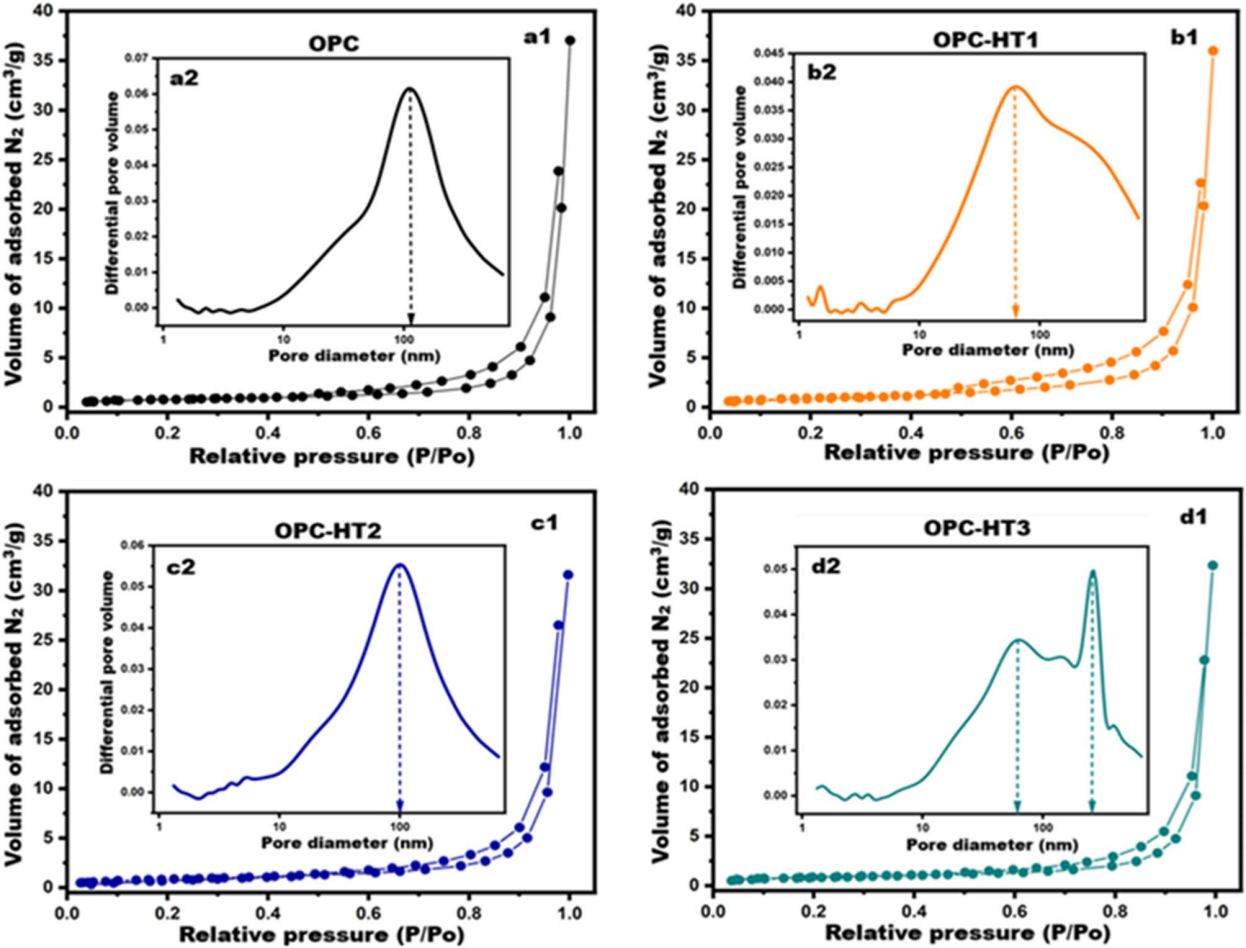
(a1-d1) adsorption/desorption isotherms and (a2-d2) BJH-pore size distribution curves for OPC-HT pastes at 28-days of curing.

It is of utmost importance to examine the porosity and textural properties of the prepared cement pastes (OPC, OPC-HT1, OPC-HT2, OPC-HT3) so that we can interpret the mechanical performance of the pastes in addition to strengthening the section related to shielding nuclear radiation. BET/BJH models were used to identify some texture parameters, such as specific surface area (SSA, cm2/g), maximum pore diameter (dpmax, nm), average pore diameter (APD, nm), and total pore volume (TPV, cm^3^/g). These textural factors reflect the actual meso-porosity of the prepared cement pastes^60,61^. Figure 7 displays N_2_-adsorption/desorption isotherms and BJH-pore size distributions for OPC, OPC-HT1, OPC-HT2 and OPC-HT3 hardened pastes at 28-days of curing. Figure 7 (a1-d1) confirms that the isotherms of all pastes are compatible with the type III/H3 hysteresis loop in which most of the pores are wide meso-pores. According to the IUPAC classification, the mesopores have a 2–50 nm average pore diameter^62,63^. Figure 7 (a2-d2) illustrates PSD-curves of the prepared pastes. The Figure indicates that the distribution curves are strongly affected by the addition of different types of HTs (HT1, HT2, HT3), as dpmax for OPC, OPC-HT1, OPC-HT2 and OPC-HT3 are 105.64, 59.50, 100 and 279 nm, respectively. Moreover, Table 5 shows all textural parameters of the fabricated pastes. The outcome confirms that the values of BJH-APD for OPC, OPC-HT1, OPC-HT2 and OPC-HT3 are 67.92, 40.23, 45.52 and 50.53 nm, respectively. These results indicate that cement paste that is free from HTs (OPC) has a large pore diameter with a macro-porous system. Meanwhile, the inclusion of 1wt.% HTs contributed to restructuring the pores of OPC-HT pastes into the meso-porous range. Moreover, OPC-HT1 possessed the highest SSA (33170 cm^2^/g), the lowest pore diameters (40.23APD-59.50dpmax, nm), and the highest total pore volume (0.045cm^3^/g). Among the three hydrotalcites, HT1 (Mg/Al = 1) demonstrated superior performance compared to HT2 and HT3 due to its unique physicochemical properties. HT1 exhibited the highest specific surface area (63.13 m²/g), greatest total pore volume (0.3209 cm³/g), smallest crystallite size (5.1 nm), and highest degree of amorphosity (approximately 60%). These characteristics enhance its adsorption capacity and catalytic activity, enabling HT1 to act as an effective nucleation site during cement hydration. As a result, HT1 facilitated the formation of additional hydration products such as CSH/Tobermorite (24.1%), Gismondite (14.3%), Lawsonite (11.6%), Hydrogarnet (5.2%), Portlandite (12.7%), and Ettringite (7.1%), as shown in Table 4. These hydration products, with diverse morphologies (needles, rods, hexagonal plates, stacked layers), progressively filled and refined the macropores of the cement matrix into a homogeneous mesoporous structure. This microstructural refinement explains the improved mechanical performance and enhanced radiation shielding observed in OPC-HT1 pastes. In contrast, HT2 and HT3, with higher Mg/Al ratios, exhibited larger crystallite sizes and lower surface areas, resulting in reduced catalytic efficiency and less pronounced pore refinement. Therefore, the Mg/Al ratio plays a critical role in determining hydrotalcite functionality, with HT1 offering the most favourable balance of amorphous structure, nanoscale size, and surface reactivity for cementitious applications^5,25,64–66^.

**Table 5:** Textural parameters for OPC-HT pastes at 28-days of curing.

Cement composite	BET surface area (cm^2^/g)	BJH average pore diameter (nm)	Max pore diameter dp_max_(nm)	Total pore volume (Vp)
OPC	27,129	67.92	105.64	0.040
OPC-HT1	33,170	40.23	59.50	0.045
OPC-HT2	30,515	45.52	100.00	0.042
OPC-HT3	28,887	50.53	276.00	0.041

### Morphology

**Fig. 8:**
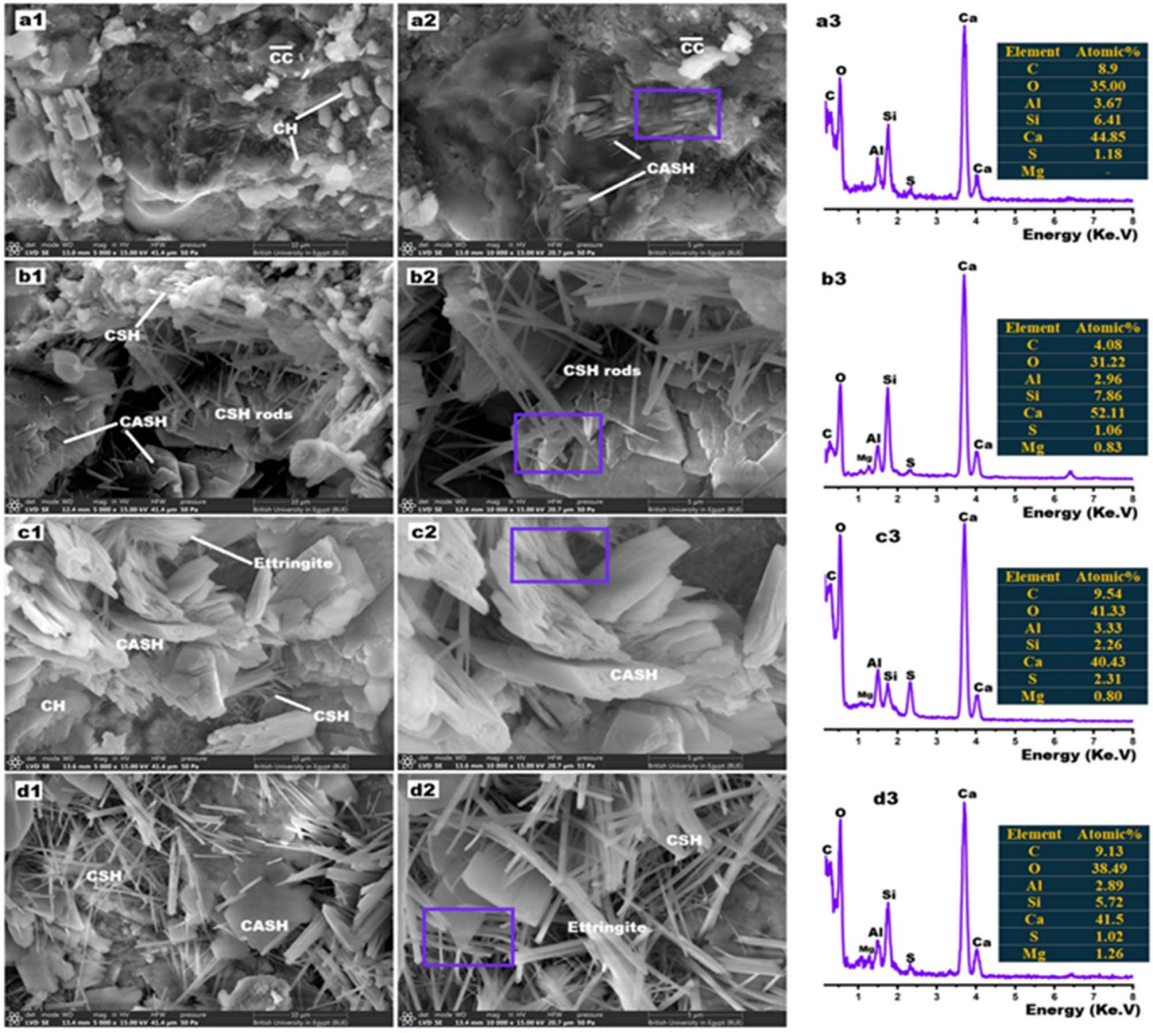
SEM/EDX images for: (a1-a3) OPC, (b1-b3) OPC-HT1, (c1-c3) OPC-HT2, and (d1-d3) OPC-HT3 pastes at 28-days of curing.

Figure 8 displays SEM/EDX images of hardened OPC, OPC-HT1, OPC-HT2 and OPC-HT3 pastes. Figure 8 (a1, a2) displays SEM images of OPC paste as a reference. Hexagonal plates of free lime (CH) and CASH (Gismondite and Lawsonite), calcite ($\:\stackrel{-}{CC}$), in addition to some unreacted grains (Larnite and Alite phases) were observed at 28-days of hydration^67^. The inclusion of 1wt.% from different types of HTs led to radical and noticeable changes in the morphological characteristics of the formed hydrates. Figure 8 (b1, b2) confirms that the inclusion of 1wt.% of highly amorphous HT1 nanoparticles induced the cement reaction to create extra rods, needles, and fibers of CSHs, hexagonal crystals of CASHs or CAH or CH, and some rods of ettringite are also present. The microstructure of OPC-HT2 contained huge quantities of stacked plates of thickened CASHs intermixed with some rods of CSHs and needles of ettringites, Fig. 8 (c1, c2). Needles of ettringite and CSHs, in addition to very thin plates of CASHs were observed in the case of OPC-HT3, Fig. 8 (d1, d2). Thus, the images indicate that the added HT has an effective role in the morphological transformations that may contribute to the development of porosity and mechanical efficiency of the pastes, with the ability to block nuclear radiation. Many previous studies have proven that adding nanomaterials (Al_2_O_3_, MgO, MgAl_2_O_4_, ZnFe_2_O_4_, Fe_2_O_3_, ZnO, NiO, TiO_2_ NPs)^24,45,68,69^ or mesoporous layered double hydroxides^47,61,70^ contributes strongly to restructuring the microstructure of doughs. Figure 8 (a3-d3) displays EDX-elemental analysis of the fabricated OPC-HT pasts. The main elements are O, C, Al, Si, Ca, S, and Mg. The absence of magnesium in cement is evidence of the absence of HT (Mg-Al-CO_3_.OH.H_2_O), but the other doughs (OPC-HT1, OPC-HT2, OPC-HT3) contain a small amount of Mg. The presence of sulfur in all pastes is evidence of the presence of ettringite (Al_2_Ca_6_H_66_O_49.68_S_3_). On the other hand, the calculated Ca/Si ratios for OPC, OPC-HT1, OPC-HT2 and OPC-HT3 are 6.99, 6.62, 17.88 and 7.25 and Ca/Al ratios are 12.22, 17.6, 12.14 and 14.34, while Si/Al ratios are 1.74, 2.65, 0.69 and 1.97, respectively. This outcome indicates that OPC-HT1 possessed the highest percentages of highly crystalline hydrogarnet (CAH) and gismondite/lawsonite (CASH), while OPC-HT2 has the highest percentage of highly crystalline CSH (Clino-Tobermorite)^71,72^. This data is in the same line with QPA analysis via the XRD technique.

### Compressive strength

**Fig. 9:**
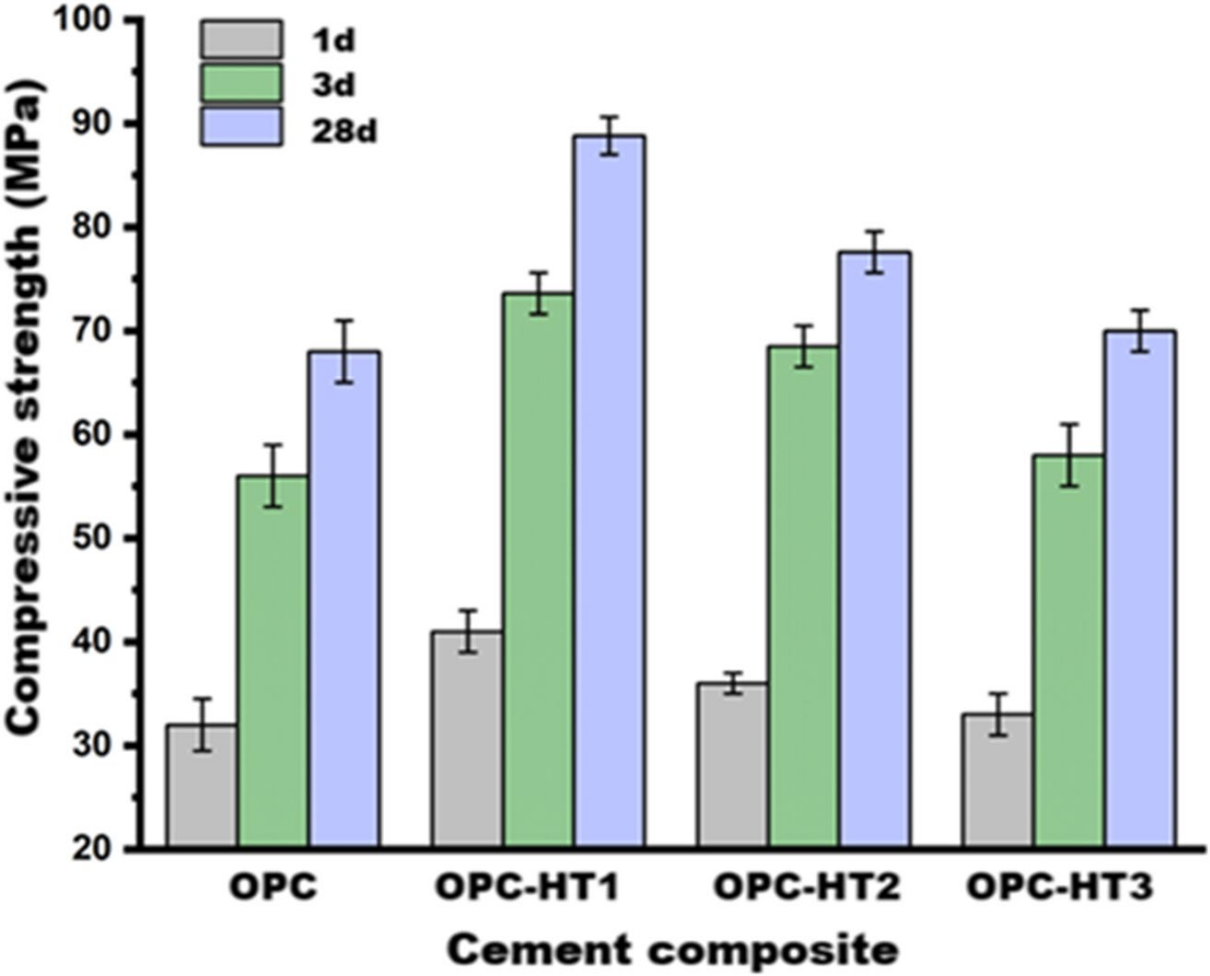
Impact of different types of hydrotalcites on the compressive strength of OPC.

The compressive-strengths of the OPC (reference specimen, 0 wt% HT), as well as OPC-HT1, OPC-HT2 and OPC-HT3 (specimens modified with 1 wt% HT1, HT2 and HT3, respectively) at 1, 3 and 28-days are represented in Fig. 9. A progression in the compressive-strengths is significantly observed with the curing time^73–75^. The compressive-strengths were increased by 112.5, 116.6, 115.6 and 112.1% after extending the curing period from 1 to 28-days for OPC, OPC-HT1, OPC-HT2 and OPC-HT3, respectively. With time, the cement particles are hydrated, forming different phases from strength-giving-phases as detected in the XRD, such as CSH (calcium-silicate-hydrate, tobermorite gel), CAH (calcium-aluminate-hydrate, hydrogarnet) and CASH (calcium-alumino-silicate-hydrate, gismondine or lawsonite)^76–78^. Increasing the amount of such phases over time filled the open pores between the cement grains, increasing the gel/space-ratio and thus increasing the strength values^79–82^.

**Fig. 10:**
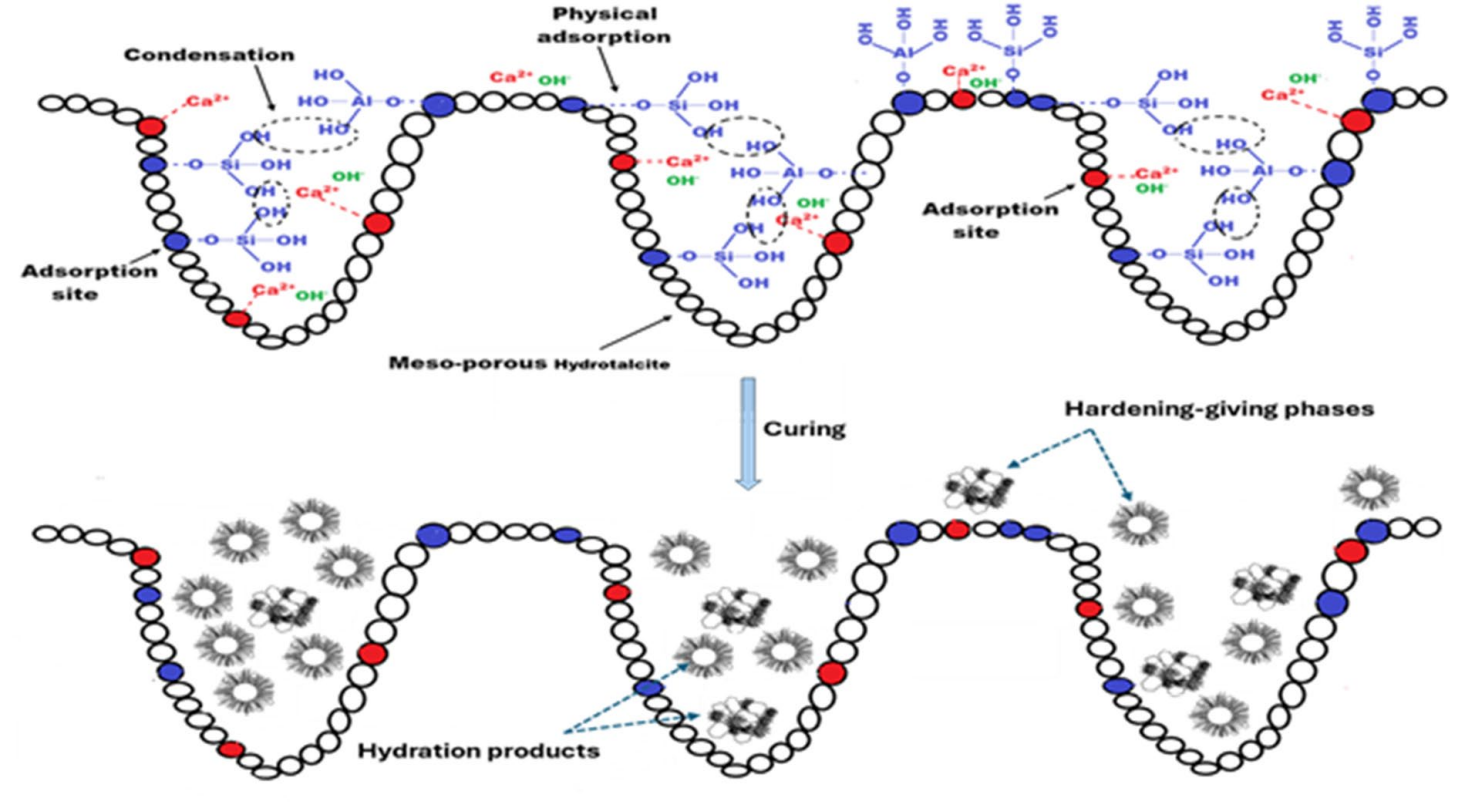
A schematic diagram clarifies the nucleation mechanism of HT compounds.

Interestingly, notice that all specimens modified with HTs have compressive-strengths higher than the reference specimen (0 wt% HT), matching with Wu et al^47^. and Chen et al^83^.. The compressive-strengths of OPC-HT1, OPC-HT2 and OPC-HT3 are higher than OPC by 28.1, 12.5 and 3.1% at early-age (1-day) and by 30.6, 14.1 and 2.9% at later-age (28-days), respectively. This improvement in the compressive strength may result from the small particle size of HTs. They can fill the pores, thus reinforcing and compacting the microstructure^48,78^. The same discussion was reported by Long et al^49,84^.; they attributed the enhancement in the compressive-strength of cementitious materials modified with calcined Mg-Al-CO_3_- based LDH to its interstitial filling that refined the pore size distribution. Furthermore, Guan et al. proved that nano Mg/Al-based HT, which is characterized by low crystallinity is thoroughly combined with the cementitious system, causing improvement in early strength^33^. The nucleation seeds mechanism that catalyzes the hydration reaction is also considered a reason for the progression in the mechanical performance^85^. After the hydration process occurs, layers of hydration products form around the unreacted particles, hindering their continuous hydration. The high surface area of HTs causes the presence of a vast number of nucleation sites in the matrix. The precipitation of hydration products on these sites decreases the thickness of the formed layers on the unreacted particles, allowing their hydration. Therefore, it accelerates the hydration process, as well as increases the amount of strength-giving-phases^65^. The nucleation site mechanism of HT compounds was previously discussed by Lauermannová et al^85^. Figure 10demonstrates a schematic diagram to describe the adsorption mechanism of hydration products on the active sites of HTs compounds. Moreover, Wu et al^47^. illustrated that the HTs’ crystal seeds reduce the energy barrier required for the precipitation of hydration products. On the other hand, HT compounds the formation of additional/secondary strength-giving-phases as the HTs provide the matrix with Al and Mg. The XRD pattern (Fig. 6) and its quantitative phase analysis (Table 4) proved that the specimens modified with HTs have a low amount of unreacted phases (alite and larnite phases) and high percentages of CAH and formation of calcium magnesium alumino oxy silicate (In OPC-HT1) compared with the reference specimen. Additionally, Wu et al^47^. reported that due to the interlayer-vacancy sites structure of HT compounds, they can capture a quantity of the mixing water, increasing the gel/space ratio^55,59^. Long et al. attributed the improvement in the compressive-strength of cementitious materials after inclusion of calcined Mg-Al-based HT to raising the pH of the pore solution, which promotes the hydrolysis of unreacted particles^84^. All these reasons reinforce the pore structure, as proved by the BET-BJH models; the average pore diameter decreased by 40.8, 32.9 and 25.6% for OPC-HT1, OPC-HT2 and OPC-HT3, concerning OPC. The smaller particle size of HT1 than HT2 and HT3 makes HT1 more effective in enhancing compressive-strength^86^.

### Radiation shielding

**Fig. 11:**
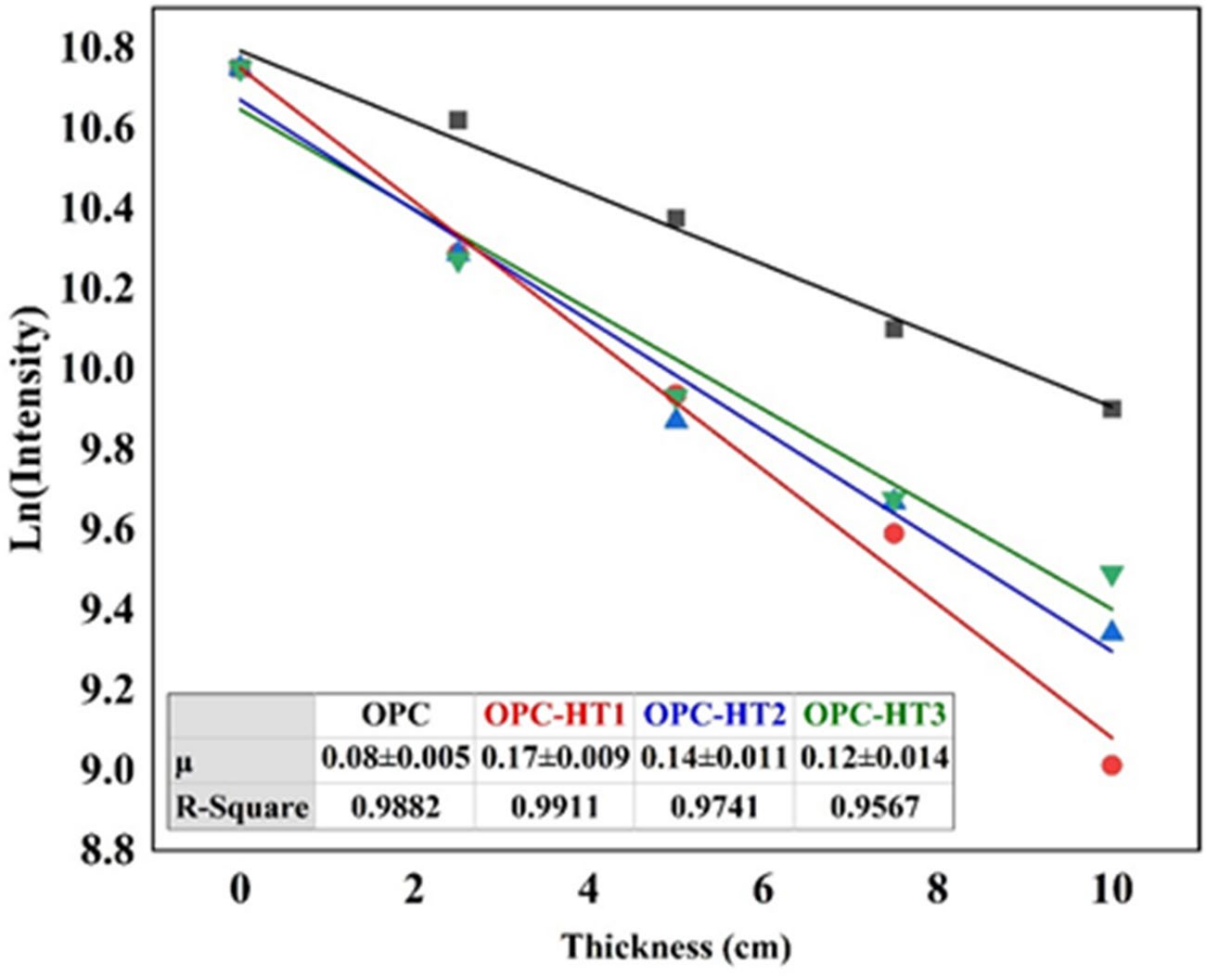
Relation between transmitted intensity and thickness of fabricated specimen.

Generally, the efficacy of materials in shielding hazards gamma-ray can be evaluated via measuring (i) the linear-attenuation coefficient (µ), that measures the materials’ ability to block radiation. The high value of µ refers to the high shielding ability; and (ii) the half-value layer (HVL) that determines the materials’ thickness required to reduce the intensity of incident radiation to half of its value. The low HVL value indicates that the small thickness of materials is able to diminish the incident radiation^57,87^. Accordingly, the µ and HVL values of specimens modified with HTs (OPC-HT1, OPC-HT2 and OPC-HT3) were measured and compared with the values of the reference specimen (OPC, 0 wt% HT) to evaluate the ability of the Mg-Al HT family in radiation shielding. The Beer-Lambert law was used to calculate the µ values for the fabricated specimens, as in Fig. 11. The data display that increasing the thickness of specimens up to 10 cm results in a substantial decline in the radiation’s intensity. According to correlation-coefficient values (R^2^ = 0.9567–0.9911), the Beer-Lambert law fits well with the obtained data.

**Fig. 12:**
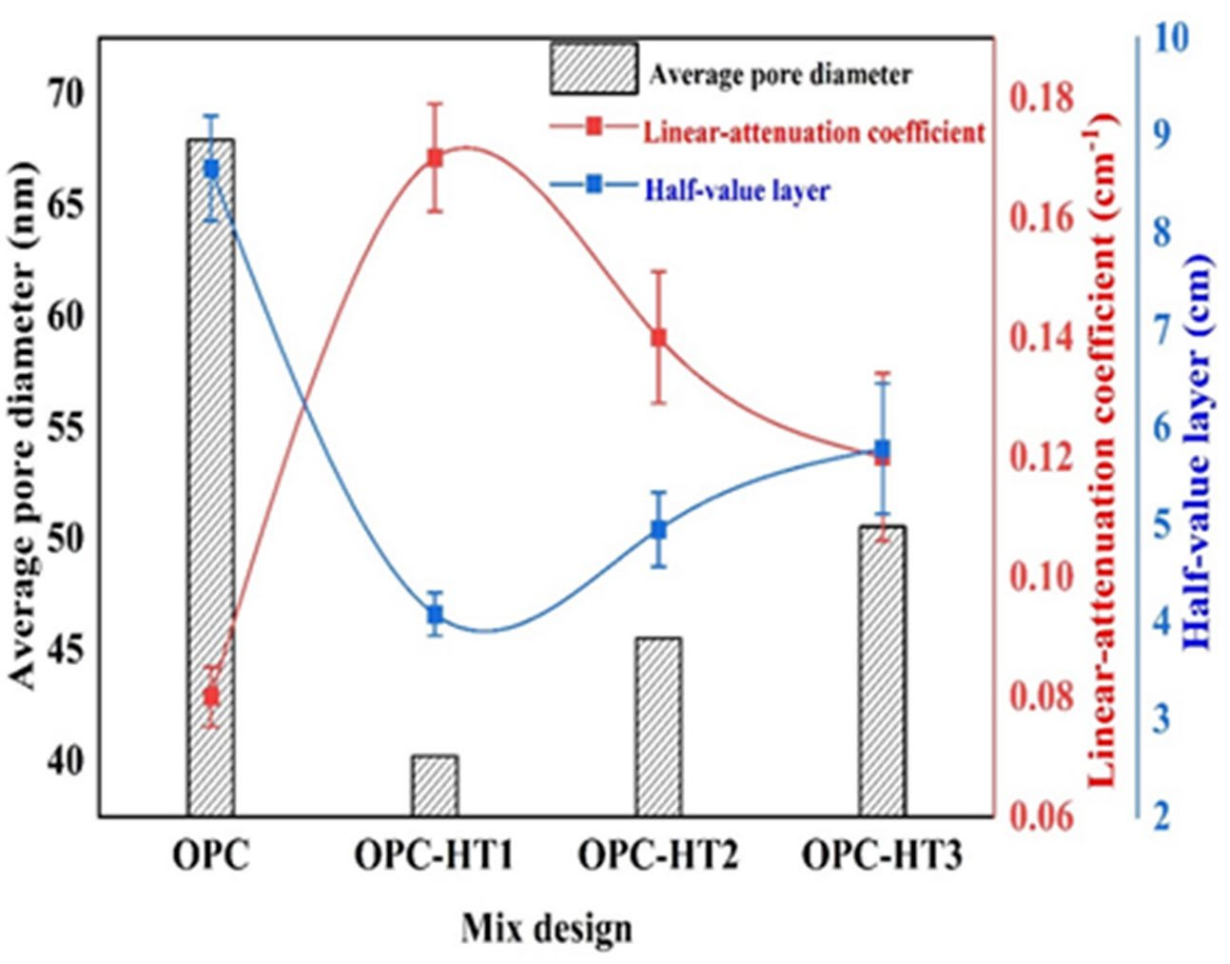
Correlation between average pore diameter, linear attenuation coefficient and half-value layer for fabricated specimen.

Figure 12 shows that OPC-HT1 has the highest µ value and the lowest HVL values (0.17 ± 0.009 cm^− 1^, 4.08 ± 0.22 cm, respectively) followed by OPC-HT2 (0.14 ± 0.011 cm^− 1^, 4.95 ± 0.38 cm, respectively), OPC-HT3 (0.12 ± 0.014 cm^− 1^, 5.78 ± 0.67 cm, respectively) and OPC (0.08 ± 0.005 cm^− 1^, 8.66 ± 0.54 cm, respectively). These data prove the high efficiency of specimens modified with HTs in gamma-ray shielding. Commonly, the ability of concrete to shield radiation depends on the type of binding materials (cement, blended-cement or geopolymer), kind of aggregate (normal or heavy-weight aggregate) and nature of additives (composition and crystallographic structure of nanomaterials or supplementary-cementitious materials)^5^. In this study, the nucleation site effect of HTs that results in forming more strength-giving phases, in addition to their filling effect, leads to compacting/densifying of the microstructure, thus hindering the transmittance of incident radiations^19,88^. Furthermore, the dosimetric nature of HTs, which is attributed to their crystallographic structure (hexagonal and rhombohedral systems, as proved in Fig. 2), makes them effective adsorbers of gamma rays, as discussed by Mohsen et al^61^. The superior efficiency of OPC-HT1 in shielding gamma rays compared to OPC-HT2 and OPC-HT3 is attributed to the effective nucleation site and filler of HT1 (which has the highest surface area concerning HT2 and HT3). These characteristics of HT1 cause the specimen modified with it to have texture parameters with the lowest pore diameter that can block radiations, as in Fig. 12.

### Scalability patterns

The scalability of HT incorporation in cementitious systems is vital for industrial adoption. HTs can be produced through cost-effective coprecipitation techniques using abundant raw materials like magnesium and aluminum salts, supporting large-scale manufacturing without major economic challenges. Additionally, the low-temperature synthesis and minimal energy use of HT preparation align with sustainable practices, cutting down on carbon emissions compared to traditional additives. From a processing perspective, HT powders can be easily mixed with cement using standard equipment, requiring no special infrastructure, which makes it easier to integrate into current production lines. However, challenges such as achieving uniform dispersion at high volumes, maintaining consistent particle size distribution, and managing moisture sensitivity during storage need to be addressed to ensure performance at scale. Furthermore, life-cycle assessments and techno-economic analyses are essential to confirm the environmental and economic advantages of HT-based formulations for broad industrial application. Overall, the potential of HTs for scalability looks promising, as long as optimized synthesis, handling, and quality control procedures are developed to support their transition from laboratory research to commercial use.

## Conclusion

Different types of hydrotalcites (HT, Mg.Al.CO_3_.OH.H_2_O) were prepared by the coprecipitation route. The molar ratio of Mg/Al was changed [1:1 (HT1), 2:1(HT2), and 3:1(HT3)] to control crystallographic structures particle sizes in addition to textural features. All synthetic HTs were individually added by 1wt.% to OPC pastes (OPC, OPC-HT1, OPC-HT2, OPC-HT3) to examine its performance in terms of fresh properties, phase composition, meso-porosity, morphology, compressive strength in addition to gamma-ray attenuation. We will present the most important points that have been reached:

- The HT of 1:1 molar ratio (HT1) possessed the highest specific surface area (63.13m^2^/g), the highest degree of amorphosity (60.39%), and a very small particle size (5.1 nm).
- Decreasing the Mg/Al ratio of HT accelerated setting times of OPC-HTs fresh pastes and caused a significant reduction in flowability.
- Quantitative phase analysis via XRD technique and SEM/EDX analyses affirmed that various types of cement hydration products (Tobermorite, Clino-Tobermorite, Gismondite, Lawsonite, Hydrogarnet, Ettringite) with different degrees of crystallinity/morphologies were created after doping 1wt.% HTs.
- Surface analysis using BET/BJH models proved that the inclusion of 1wt.% HT1 to OPC pastes contributed to restructuring the pores into the mesoporous range with the highest SSA (33170 cm^2^/g) and the lowest average pore diameter of 40.23 nm.
- Adding HTs enhances the radiation shielding of the cement matrix. OPC-HT1 achieved the highest linear-attenuation coefficient and lowest half-value layer owing to its compact structure and dosimetric characteristics of HT1.
- HTs can be introduced as sustainable cement additives that boost strength, durability, and radiation shielding while lowering carbon footprint, enabling greener and safer construction.

## Data Availability

All data generated or analysed during this study are included in this published article.
